# Neurological Phenotype of Mowat-Wilson Syndrome

**DOI:** 10.3390/genes12070982

**Published:** 2021-06-27

**Authors:** Duccio Maria Cordelli, Veronica Di Pisa, Anna Fetta, Livia Garavelli, Lucia Maltoni, Luca Soliani, Emilia Ricci

**Affiliations:** 1 IRCCS Istituto delle Scienze Neurologiche di Bologna, UOC Neuropsichiatria dell’Età Pediatrica, 40139 Bologna, Italy; veronica.dipisa@aosp.bo.it (V.D.P.); anna.fetta@studio.unibo.it (A.F.); lucia.maltoni3@unibo.it (L.M.); luca.soliani2@unibo.it (L.S.); 2 Medical Genetics Unit, Department of Mother and Child, Azienda USL-IRCCS di Reggio Emilia, 42123 Reggio Emilia, Italy; livia.garavelli@ausl.re.it; 3 Child Neuropsychiatry Unit, Epilepsy Center, San Paolo Hospital, Department of Health Sciences, University of Milan, 20142 Milan, Italy; emilia.ricci85@gmail.com

**Keywords:** *ZEB2*, neural crest, GABAergic transmission, corpus callosum, epilepsy, sleep disorders, neurodevelopmental delay, intellectual disability.

## Abstract

Mowat-Wilson Syndrome (MWS) (OMIM # 235730) is a rare disorder due to *ZEB2* gene defects (heterozygous mutation or deletion). The *ZEB2* gene is a widely expressed regulatory gene, extremely important for the proper prenatal development. MWS is characterized by a specific facial gestalt and multiple musculoskeletal, cardiac, gastrointestinal, and urogenital anomalies. The nervous system involvement is extensive and constitutes one of the main features in MWS, heavily affecting prognosis and life quality of affected individuals. This review aims to comprehensively organize and discuss the neurological and neurodevelopmental phenotype of MWS. First, we will describe the role of *ZEB2* in the formation and development of the nervous system by reviewing the preclinical studies in this regard. *ZEB2* regulates the neural crest cell differentiation and migration, as well as in the modulation of GABAergic transmission. This leads to different degrees of structural and functional impairment that have been explored and deepened by various authors over the years. Subsequently, the different neurological aspects of MWS (head and brain malformations, epilepsy, sleep disorders, and enteric and peripheral nervous system involvement, as well as developmental, cognitive, and behavioral features) will be faced one at a time and extensively examined from both a clinical and etiopathogenetic point of view, linking them to the *ZEB2* related pathways.

## 1. Introduction

Mowat-Wilson Syndrome (MWS) (OMIM # 235730) is a condition due to *ZEB2* gene defects (heterozygous mutation or deletion) [1] and is characterized by a wide clinical spectrum that ranges from mild (usually associated with missense mutations) to severe forms [2].

It is a rare syndrome: in the literature a little less than 300 patients have been described so far [3,4,5,6,7,8,9,10,11,12,13,14,15]. In 2018, Ivanovski et al. [7] described the phenotype and genotype of MWS, analyzing for the first time a large population (87 patients). In 2020, specific MWS growth charts were published [16].

Patients with MWS usually display a specific facial gestalt with, in particular, high forehead, uplifted earlobes with a central depression, rounded nasal tip with prominent columella and broad nasal bridge, hypertelorism, deep-set eyes, strabismus, medial flaring of the large eyebrows, prominent triangular chin, and widely spaced and/or malpositioned teeth with dental crowding [7,15].

Musculoskeletal anomalies can be various; the most frequent are slender and tapering fingers, mild calcaneovalgus, long toes, pes planus and scoliosis. Congenital heart diseases are also characteristic of this syndrome (60.5% of patients), especially patent ductus arteriosus, atrial and ventricular septal defects, pulmonary stenosis, and aortic coarctation. Eye involvement could be present with strabismus (56.8%), astigmatism, and myopia. Other clinical aspects associated with MWS are constipation (43.5%), Hirschsprung disease (30.6%), urogenital anomalies (61.25%; in particular hypospadias and cryptorchidism), and short stature [7].

Despite the multiple congenital anomalies mentioned before, the main feature of MWS is the neurologic and neurodevelopmental involvement. These aspects could heavily condition prognosis and life quality in patients with MWS [17]. In this review, we will specifically analyze the neurological and neurodevelopmental phenotype in MWS focusing also on *ZEB2* function in brain development.

## 2. *ZEB2* and Nervous System Development

The *ZEB2* gene synthesizes a zinc finger protein that binds to DNA regulating the expression of several specific genes [18,19,20]. Its functions can be different (repression or activation of gene transcription) depending on the binding site and on the presence of enhancers in the distal regions of the gene, which can vary depending on tissues [21]. *ZEB2* has a core function in organisms’ development and is highly conserved in different species [22]. In particular, this gene takes part in the embryological development of the neural tube (primordial central nervous system) and neural crest (primordial peripheral nervous system) [23,24,25,26,27]. Indeed, *ZEB2* knockout mouse embryos prematurely die due to impairment in the development of these structures [28,29]. Moreover, *ZEB2* plays a key role also in the subsequent stages of nervous system development.

In the telencephalon, it represses Sfrp1 transcription, reducing the extracellular inhibitor of Wnt signaling Secreted Frizzled-Related Protein 1 [30]. Mouse embryos with conditional *Zeb2* knockout in dorsal telencephalic cortical progenitors show an increase in Sfrp1 transcription with greater inhibition in Wnt signaling, especially in hippocampal regions. This leads to reduced neuronal progenitor proliferation and to increased apoptosis in these regions with a consequent absence of hippocampal and corpus callosum structures [31].

In the embryologic neocortex, *ZEB2* is highly expressed and represses Nft3 and Fgf9 expression. Mouse embryos with conditional *Zeb2* knockout in the neocortex show increased expression of these two genes that leads to a greater development of neocortical glial precursors in the embryologic phase and to increased astrocytogenesis in the post-natal phase [32]. Moreover, in the neocortex, *ZEB2* regulates also axonal projections’ development with the upregulation of the microtubule binding protein Ninein [33] and the repression of the inhibitor of axonal growth BMP7 [34]. Mouse embryos with conditional *Zeb2* knockout have a downregulation in Ninein expression and an upregulation in BMP7 expression. This leads to an impairment in the development of axonal projections with consequent reduced or absent corpus callosum, commissure, and corticospinal tract [33]. Furthermore, *ZEB2* represses Nkx2.1 expression in the medial ganglionic eminence, promoting the development of cortical GABAergic interneurons and repressing that of striatal GABAergic interneurons. Mouse embryos with conditional *Zeb2* knockout in the medial ganglionic eminence have an imbalance in the development of GABAergic interneurons, which are reduced in the cortex and increased in striatal regions [35,36]. These changes in interneuron development reduce cortical inhibition and seem to be related to increased susceptibility to epilepsy [37]. *ZEB2* also represses the inhibitors of BMP-Smad activated expression of differentiation, leading to oligodendrocyte differentiation and normal cerebral myelination. Mouse embryos with conditional *Zeb2* knockout in the neocortex have increased inhibitors of this way with reduced cerebral myelination [38].

In the cerebellum, *ZEB2* plays a central role in Bergmann glia development, but is not expressed in cerebellar astrocytes. Mouse embryos with *Zeb2* knockout in cerebellar radial glia display dysgenesis in cerebellar cortical lamination with impairment in proliferation and migration of glial precursors and in differentiation of Purkinje cell layer [39]. This leads to movement deficit in mice and could also explain locomotor difficulties in MWS.

Moreover, *ZEB2* also has a central role in the development of the peripheral nervous system, both in neural crest and in its derivatives (Schwann cells, sensory neurons, enteric nervous system, melanocytes) [28,29].

In neural crest, *ZEB2* plays a role in regulating the endothelin–endothelin receptor signaling system. An activation of endothelin receptor B (EDNRB) causes a reduced maturation of Schwann cells and enteric nervous system from their precursors in neural crest [40,41,42].

In Schwann cells, *ZEB2* represses the expression of Sox2, which has a key role in inhibiting Schwann cells differentiation and myelination. Indeed, mutant mice with *Zeb2* knockout in Schwann cells have a persistent Schwann cells proliferation and repressed peripheral nerve myelination [41,43]. In Schwann cells, *ZEB2* also represses Hairy/enhancer-of-split related with YRPW motif protein 2 (Hey2) activity that contributes to Schwann cells differentiation [41]. In dorsal root ganglion, *ZEB2* has a core function in the development of sensory neurons for nociceptive fibers [44] activating the Neurog1-dependent neurogenesis [45]. Mice with conditional *Zeb2* knockout in these regions have reduced or absent pain sensitivity due to reduced nociceptor differentiation from neural crest precursors [45].

In the ventral spinal cord, *ZEB2* contributes to visceral motor neuron development. Indeed, *ZEB2* together with Sox10 represses the BMP signaling expression that reduces the enteric neural crest cells’ migration and influences the orientation of nervous fibers [46]. Mice with conditional *Zeb2* knockout in these regions have a reduced development of visceral motor neurons [47], which leads to Hirschsprung disease.

In melanocytes, *ZEB2* plays a role in their differentiation from melanoblasts activating Microphthalmia-associated transcription factor (Mitf) [48]. Mutant mice show melanocytes that are not properly differentiated and depigmented hair follicles [48].

As demonstrated above, *ZEB2* has a central function in the development of the central and peripheral nervous system. However, several mechanisms of action are still unclear or unknown and further studies are required to clarify these aspects. Moreover, to create a mouse model of Mowat-Wilson Syndrome, it is necessary to have a conditional *Zeb2* knockout of both alleles, otherwise in the human species an inactivated allele is sufficient to manifest the disease. This is probably due to a human increased susceptibility to *ZEB2* low dosage [49]. In the future, a deeper knowledge of these aspects could be useful to develop targeted therapies for epilepsy and for reduced pain sensitivity [2,49].

**Figure 1:**
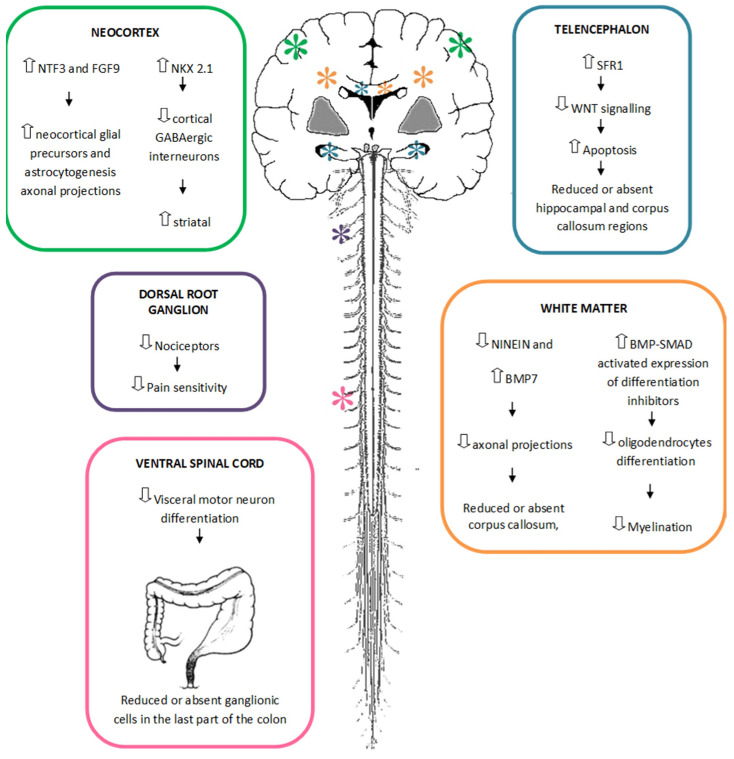
Role of *ZEB2* haploinsufficiency in central and peripheral nervous system development.

In Figure 1, we tried to summarize all the aspects mentioned above.

### Genotype/Phenotype Correlation of ZEB2 Mutations

To date, no solid genotype/phenotype correlations have been found in MWS; however, the paper which described the largest cohort of MWS individuals suggested some interesting considerations [7]. Firstly, the deletions encompassing the whole gene were associated with more severe clinical manifestations, including a worse neurodevelopmental delay. This was probably due to contiguous genes and/or noncoding RNAs involvement. Secondly, the authors described two patients with intragenic variants resulting in the synthesis of a defective protein without typical haploinsufficiency effect that presented a mild phenotype (no epilepsy and mild-moderate intellectual disability in one case); however, some patients with the abolished protein had a mild clinical presentation too. No patients with missense mutations were enrolled in this study [7]. Some case reports of patients with *ZEB2* missense mutations were described [50,51,52], reporting different, usually mild, neurological and electroclinical phenotypes but without univocal conclusions. Further studies are needed to better investigate genotype/phenotype correlations in MWS, especially in terms of neurological manifestations.

## 3. Neurological Involvement of MWS

The role of *ZEB2* in nervous system development results in severe neurological involvement in humans with Mowat-Wilson Syndrome due to *ZEB2* haploinsufficiency. Indeed, individuals with Mowat-Wilson Syndrome display microcephaly, brain malformations, epilepsy, sleep disorders, and cognitive and behavioral impairment.

### 3.1. Head and Brain Malformations

Individuals with *ZEB2* deletions or intragenic mutations have a spectrum of cranial and brain anomalies reported since the first descriptions of the syndrome [12,15,53,54,55].

Microcephaly is extremely common, reflecting the known influence of *ZEB2* on neural crest and neural tube development [2,24,49], with its frequency established at around 84% by Mowat et al. [53]. Ivanovski et al. demonstrated its mostly secondary nature, with minimal deviation from the reference values at birth in both genders, an increasing deviation between 2 months and one year of life, and then a flattening of the curve until 16 years of age, when it reaches <3rd percentile/<−2SD for both females and males [16].

Craniosynostosis has been reported in two patients [56,57].

Garavelli et al. analyzed the neuroradiological features of a cohort including 54 patients with molecularly confirmed *ZEB2* haploinsufficiency and compared them with literature data, outlining a neuroradiological phenotype of the syndrome [58].

Consistent with the role of the transcription factor *ZEB2* in regulating intracortical and cortical-subcortical connections [33], the corpus callosum abnormalities turn out to be a hallmark of brain MRI findings in these individuals, presenting in about 75% of patients. Garavelli et al. reported complete agenesis of the corpus callosum in about 25% of individuals with MWS, partial agenesis in 16%, and callosal hypoplasia in 37% [58].

A significantly higher incidence of complete agenesis of the corpus callosum in patients with predicted synthesis of a defective protein than in those with a complete absence of the protein has been found, suggesting a dominant-negative effect from protein accumulation on corpus callosum formation.

Another frequent hallmark of MWS is hippocampal malformations. Although previously reported in a minority of patients, in more recent and focused studies, bilateral hippocampal morphological or positional abnormalities have been found in up to 77.8% of patients [58].

As mentioned before, *ZEB2* is essential for hippocampus development, indirectly acting as a positive Wnt regulator whose signaling likely decreases proliferation of neuronal progenitors and increases apoptosis of postmitotic neurons in the hippocampus and dentate gyrus, as observed in *Zeb2* mutant mice [31].

White matter abnormalities are also common, including reduced thickness in almost half of MWS individuals; localized signal alterations of white matter are less frequent [58]. *ZEB2* is an essential modulator of central nervous system (CNS) myelination repressing differentiation inhibitory signals while activating crucial oligodendrocyte-promoting factors such as Smad7 [38]. Ventriculomegaly often co-occurs, with a widening of the temporal horns or a lateral ventricle enlargement [12,58].

Malformation of cortical development such as polymicrogyria, pachygyria, periventricular heterotopia, and focal cortical dysplasia may be observed in a few patients [55,58,59]; these cortical malformations are supposed to be related to *ZEB2* role in neurogenesis [60].

Rare cerebellar involvement has been reported (hypoplastic or macrocerebellum, absent or small cerebellar vermis) [55,58], likely related to the role of *ZEB2* in inducing granule neuron progenitors’ migration, glial precursors’ proliferation, and radial glia differentiation into Bergmann glia in the Purkinje cell layer [39].

Other rare reported findings are large basal ganglia (in this regard, it is interesting to mention the recently discovered role of *ZEB2* in the formation and migration of dopaminergic neurons [61]) and Chiari type 1 malformation [15,58].

The occurrence of a CNS tumor is possible although uncommon [58,62].

### 3.2. Epilepsy

**Figure 2:**
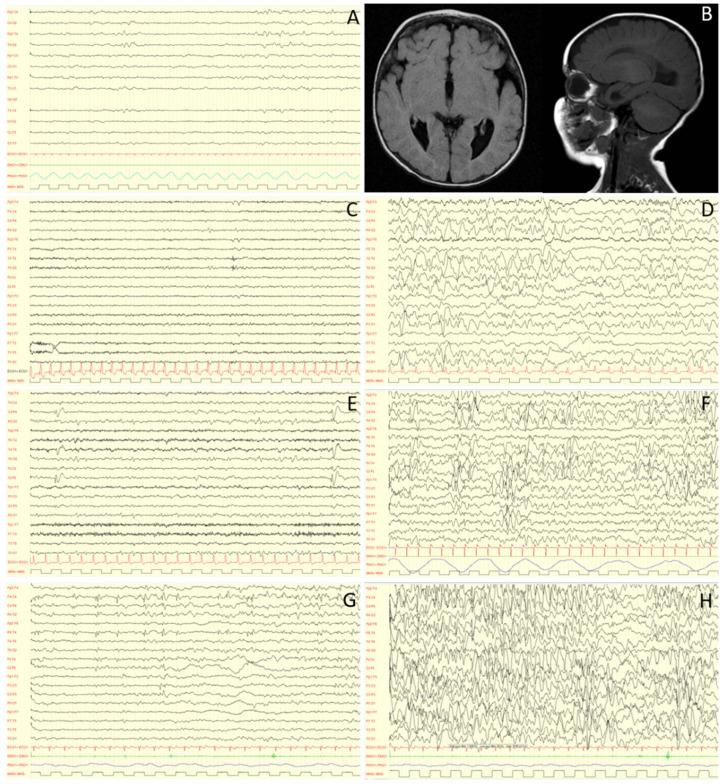
EEG evolution at 0 (**A**), 3 (**C**,**D**), 5 (**E**,**F**) and 9 (**G**,**H**) years old, and RM features (**B**) of a MWS individual. Caption: At birth, electroencephalographic activity is essentially normal (**A**). With time, a worsening is observed, with an increase in paroxysmal abnormalities in wakefulness (**C**,**E**,**G**), dramatically activated during sleep (**D**,**F**,**H**). MRI images performed at 4 months show complete agenesis of the corpus callosum (**B**). Interestingly, abnormalities in sleep can sometimes appear asynchronous although bilateral: this is probably related to this type of malformation. Note: low frequency filter: 1.6 Hz; high frequency filter: 60 Hz; paper speed: 20mm/sec; sensitivity: 150 microV/cm (**A**,**C**,**D**,**E**,**F**), 200 microV/cm (**G**) and 100 microV/cm (**H**).

Epilepsy is a major feature of both MWS and *ZEB2* mutations. The prevalence of epilepsy in MWS has been described with a mean of about 75–80% in the most recent series [7,17]. The electroclinical phenotype was specifically investigated in a previous paper including a cohort of 22 patients, the largest sample ever described regarding this topic [17]. In this study, Cordelli and colleagues described for the first time some specific electroclinical features of the syndrome [17]. In the majority of patients, epilepsy starts with focal seizures, triggered by fever, during the first years of life. Later, focal seizures continue not precipitated by fever. The semeiology may be different: hypomotor, versive, and focal clonic with a range from daily to sporadic in terms of frequency [17]. Then, atypical absences also often occur starting from 4 years of age and are sometimes difficult to recognize. Frontal regions appear as the most involved areas in terms of epileptogenic zones [17,63]. An age-dependent evolution has also been highlighted regarding electroencephalography (EEG) features [17] (Figure 2): earlier in life, the EEG background activity (asleep and awake) is normal and there are no paroxysmal discharges, while later in life, a slowing in background activity appears together with focal and multifocal high-voltage spikes and spike-waves prevailing in the frontal and central areas. These epileptic discharges show relevant activation during sleep, delineating a continuous or nearly continuous spike and wave activity pattern in several patients [63,64]. An age-dependent EEG sleep pattern was confirmed also by Di Pisa et al. [64]: all patients showed delta waves of background activity during sleep with a poverty of physiological sleep figures (spindles and K-complex), mainly in the first hours of the night. In younger children, they reported only isolated focal spikes minimally activated by sleep. In older children (>6 years), they observed in NREM EEG subcontinuous to almost continuous diffuse frontal predominant discharges of spike-waves, especially in the first sleep cycle, often constituting electrical status epilepticus during sleep (ESES) [64]. ESES was described also by Bonanni et al. in a case series study (7 patients) outlining its possible impact on cognitive impairment; an improvement after steroid treatment was reported [63]. However, no conclusive results are available about this topic, and to date, it is very difficult to assess the exact ESES role on the severity of the patients’ intellectual disability.

No unequivocal data are available about the prognosis of epilepsy and the number of drug-resistant patients; however, seizures are surely challenging to treat in more than half of the patients requiring polytherapy [7,17,63]. To date no solid data are available regarding the best therapeutic approach and the long-term evolution of epilepsy. Concerning antiepileptic drugs, valproic acid is reported as the most used and effective both for focal seizures and absences [17]. As regards non-pharmacological treatment, only one case report describes partial response after vagus nerve stimulator (VNS) implantation [65]. It is important to note that the only results about epilepsy treatment are from observational studies and detailed data are lacking about better drug associations and follow-up of seizure-free patients. A very recent paper describes the management of drug-resistant status epilepticus in an adult patient with MWS, finally treated with valproic acid, levetiracetam, and zonisamide in association [66]. The incidence of status epilepticus has not been investigated yet, but its treatment may be a challenge for clinicians.

As regards epilepsy etiology, the first observations have suggested a structural origin of seizures, with focus on hippocampal abnormalities, corpus callosum hypo-/agenesis, and microcephaly [67]. Then, a genetic etiology, independent of cerebral malformation, has been suggested and confirmed over the years. The GABAergic interneurons appear to have a major role. Indeed, a lot of different papers demonstrate the important role of *ZEB2* in both GABAergic interneurons’ migration and differentiation in mice models [2,35,36]. Thus, the result of *ZEB2* mutations in patients with MWS may be the imbalance between GABAergic cortical/subcortical interneurons, which probably promote and sustain the spreading of dysregulated excitatory post-synaptic potentials leading to EEG discharges and seizures [37,63]. Moreover, ZEB2 has been shown to play a key role in different neurodevelopmental steps including neural tube and neural crest formation, gliogenesis, differentiation of the hippocampal/neocortical pyramidal neurons, and regulation of midbrain dopaminergic neurons [2]. Interestingly, over the last years, some case reports have described different epileptic phenotypes associated with ZEB2 missense mutations in patients with no clinical features of MWS [68,69].

Further studies are needed to better define epilepsy and its evolution throughout the patients’ lifetime, as well as considering its important impact on the quality of life.

### 3.3. Sleep Disturbances

Sleep disorders are often reported in Mowat-Wilson Syndrome (MWS).

Evans et al. studied sleep disturbances with the administration of the “Sleep Disturbance Scale for Children” (SDSC) questionnaire [70]. They found a high level of sleep disturbance in MWS patients, with the highest scores on the Sleep–Wake transition disorders subscale (91% of patients reaching at least the borderline score). These results were confirmed in a following paper about a sleep clinical study in which the analysis of the SDSC questionnaire reported high scores for the “Sleep-wake transition” and in “Initiating and maintaining sleep” [64]. In the same article, Di Pisa et al. objectively evaluated the sleep pattern in the MWS sample through a video-polysomnographic study [64]. The results of this study allowed for a better definition of sleep disorders in MWS and correlated well with the findings of the questionnaire. In regard to sleep architecture, they observed significant differences among sleep parameters of controls and MWS patients, particularly a significant increase in WASO and arousal index and a reduction in TST. These data did not seem to be related to age or influenced by respiratory or frequency EEG abnormalities. They suggested that SDSC could be considered an instrument to evaluate and monitor sleep in the syndrome [64]. Both studies emphasized the importance of screening for sleep disorders and their potential treatment, such as melatonin, benzodiazepines, or niaprazine [64,70].

Di Pisa et al. also highlighted an age-dependent EEG sleep pattern, which is described in the epilepsy section [64].

About pathogenetic hypotheses, a GABAergic mechanism was proposed for sleep-related EEG abnormalities, as in other genetic syndromes with neurodevelopmental disability and epilepsy, such as Angelman syndrome [71]. Moreover, *ZEB2* is crucial for the formation of intracortical, intercortical, and cortico-subcortical connections [2,33]. Alterations of brain connectivity could explain, at least in part, the sleep disturbances observed in MWS individuals.

Head and brain malformations, epilepsy, and sleep disorders are summarized in Table 1.

### 3.4. Enteric and Peripheral Nervous System Involvement

Enteric nervous system involvement in MWS has been reported since the first description of the disease in 1998 [73].

Later studies confirmed that Hirschsprung disease was present in several children with MWS. Moreover, constipation without Hirschsprung disease appears to be present in an additional number of cases, thus representing an important issue in these individuals [13,54].

Recently Dagorno and colleagues described the functional outcome of 10 individuals with MWS associated to Hirschsprung Disease. In this group of patients, a high rate of immediate surgical complications has been noted, but some patients may achieve a bowel function comparable with non-syndromic HD patients, suggesting the importance of surgical intervention in this population [74].

*ZEB2* has been demonstrated to play a central role in the development of the peripheral nervous system both in neural crest and in its derivatives (enteric nervous system, Schwann cells, sensory neurons, melanocytes) [28,29].

*ZEB2* specifically contributes to visceral motor neuron development. Mice with conditional *Zeb2* knockout in these regions have a reduced development of visceral motor neurons [47], which could lead to Hirschsprung disease.

Underreaction to pain is a frequently reported symptom in MWS patients. It was reported in 44 over 67 patients in the cohort reported by Ivanowski et al. [7].

In the largest study to date about the behavioral phenotype in MWS, underreaction to pain was reported in about 60% of patients. In this study, it was not possible to distinguish between insensitivity to pain, which can reflect an involvement of pain sensation pathways, and pain indifference, which is not associated to peripheral nerve abnormalities since this symptom was investigated only with the Developmental Behaviour Checklist (DBC) questionnaire [75].

Pathogenic hypothesis are related to the *ZEB2* function in the development of sensory neurons for nociceptive fibers in dorsal root ganglion [44], activating the Neurog1-dependent neurogenesis [45]. Mice with conditional *Zeb2* knockout in these regions have reduced or absent pain sensitivity due to reduced nociceptor differentiation from neural crest precursors [45].

### 3.5. Developmental and Cognitive Aspects

To date, knowledge about developmental and cognitive characteristics in Mowat-Wilson syndrome is generally limited to case reports, case series, and review articles [3,4,7,8,9,10,12,53,63,73,75,76,77,78,79,80]. In many of the reports, the exact values of cognitive level and the tests used are not reported.

Developmental delay is usually observed since the first months of life, with hypotonia frequently noted as one of the first symptoms. In the cohort described by Ivanovski and colleagues [7], it was present in 79.1% of patients. Developmental milestones such as sitting and walking are very delayed (in the same cohort, mean age of sitting without support is 19.39 months, and mean age of walking is 3 years and 9 months); though some remain non-ambulatory, some patients could show ambulation with a wide-based or ataxic gait, thus reminiscent of individuals with Angelman syndrome. Fine motor skills and adaptive skills are usually impaired [7].

Speech in MWS patients is usually impaired, with some patients that cannot speak or with language limited to few words. In many patients receptive language skills seem to be preserved [76]. Considering that, it is advisable to use augmentative and alternative communication (AAC) in patient care and rehabilitation to improve the communication skills of MWS patients [7].

Regarding cognitive aspects, Evans et al. reported the cognitive level in 61 patients, and even though it was not possible to use a single type of IQ assessment, all patients were described as having a severe to profound intellectual disability (ID) [75]. In the paper of Ivanovski et al., describing the clinical phenotype of 87 patients with MWS, almost all the patients are described with moderate to severe ID [7]. Isolated cases with an exceptionally mild phenotype have been reported by Zweier et al. [80] in a 5-year-old child and by Yoneda et al. [78], but the exact cognitive IQ levels were not reported. Bonanni and others [63] reported, in 5 patients, electrical status epilepticus during sleep, speculating that there was a possible effect of this condition on intellectual, motor, and behavioral functioning in MWS patients.

*ZEB2* has been demonstrated as having a role in the development of many SNC parts, such as regulating cortical-subcortical connections, myelination, and hippocampal development. Defects in the formation of neocortical axonal projections have been demonstrated in *Zeb2* knockout [32,33,81]. Taking such neocortical defects together with those described by Miquelajauregui et al. [31], these *Zeb2*-deficient mouse models could explain the severe mental retardation observed in MWS patients. *ZEB2* is a key regulator for Bergmann glia formation involved in cerebellar development, and *ZEB2* loss causes cerebellar foliation defects, thus contributing to motor deficit in MWS [39].

### 3.6. Behavioral and Psychopathological Aspects

In the first reports of the syndrome, patients with MWS were reported as having a happy demeanor with frequent smiling [9,53,73], and persons with MWS are usually described as having a ‘‘generally happy, social personality’’ [8] or as ‘‘socially engaging and responsive’’. Following these reports, the role of *ZEB2* in mood and a potential link between the rat homolog of *Zeb2* and antidepressant function have been proposed [61,69].

More recently, Evans and colleagues [75] have described the behavioral phenotype of patients with MWS administering the Developmental Behavioral Checklist to caregivers of 61 individuals and compared the results with a control group of individuals with ID from various etiologies. In contrast with previous data, inappropriate laughter appeared to be no more common in MWS individuals than in those of similar age and level of ID [75]. In this group, overall rates of clinically significant psychopathology and behavioral and emotional problems in MWS patients were similar to rates of the general ID population [75].

In another study by Niemczyk [82], symptoms of incontinence and behavioral problems have been investigated by the administration of questionnaires to parents and caregivers. Behavioral symptoms were reported in 39.1% of individuals with MWS using the Developmental Behaviour Checklist (DBC).

In the cohort reported by Ivanovski [7], some behaviors appeared to be more frequent, such as mouthing or chewing objects or body parts and bruxism. Other behavioral patterns, such as laughing for no obvious reason, unrealistic happiness or elation, switching lights on and off, rapid mood changes, standing close to others, and eating nonfood items, were also common.

Some patients with MWS also exhibit motor stereotypies like repeated movements of hands and head; other children are reported as fascinated by turning the pages of books and magazines [12]. In the study published by Evans and colleagues [75], stereotyped behaviors were noted to be more frequent with a higher score for the items ‘‘flicks taps twirls objects’’ (DBC item 25) and ‘‘switches lights on and off or similar repetitive activity’’ (DBC item 72) reported by other authors.

To date, there is only one report that describes the psychopharmacological management of behavioral problems in MWS [83]. The authors reported the need for association of antiepileptic drugs with antipsychotic drugs in patients with MWS to manage behavioral problems when associated with epilepsy. They also reported an adverse effect to psychostimulants, thus recommending to use these drugs with caution [83].

Interestingly, Ripke et al. [84] reported a significant association between specific SNP (rs12991836) located near the *ZEB2* gene and schizophrenia in Caucasian patients. In another study by Khan [85], case–control analysis was designed to investigate whether common SNPs covering the *ZEB2* gene were not only associated with schizophrenia but also with bipolar disorder and mood dysregulation disorder; finally, rs6755392 was found to be significantly associated with schizophrenia.

GABAergic interneurons seem to play an important role in synchronizing brain activity in distinct regions of the brain, and abnormalities in these interneurons may be the cause of psychosis [86]. Thus, it could be suggested that *ZEB2* is a contributing factor affecting GABAergic cortical interneurons for the subsequent development of schizophrenia. However, no cases of schizophrenia have been observed so far in individuals with MWS.

Finally, in the cohort reported by Ivanovski [7], over 60% of patients (42 of 65) showed underreaction to pain, which can be dangerous for these children. Underreaction to pain results from reduced responsivity to nociceptive stimulation rather than an inability to communicate discomfort. *ZEB2* has been demonstrated to have a core function in the development of sensory neurons for nociceptive fibers in the dorsal root ganglion. Murine models with conditional *Zeb2* knockout in these regions have reduced or absent pain sensitivity [44].

Behavioral and psychopathological aspects are summarized in Table 2.

## 4. Future Perspectives

To date, the neurological phenotype has been specifically described in few papers in the literature. Laboratory research has been providing very important hypotheses about the pathogenic role of *ZEB2* mutations in both prenatal and postnatal neurodevelopment. Some important correlations among epilepsy, cognitive impairment, behavior, sleep patterns, and quality of life need to be investigated in the next years in order to target the treatment. In this regard, clinical data about long-term evolution would be beneficial as they may represent the first next step to evaluate the overall clinical evolution over patients’ lifetimes.

## Figures and Tables

**Table 1:** Main neurological features in Mowat-Wilson Syndrome.

Neurological Features			Possible Therapeutic Intervention
head and brain malformations	head size and skull	microcephaly [2,24,49,53]	
		Craniosynostosis [56,57]	neurosurgery [56,57]
	corpus callosum	complete agenesis [58]	
		partial agenesis [58]	
		hypoplasia 37% [58]	
	hippocampus	bilateral morphological abnormalities or positional anomalies [58]	
	white matter abnormalities	reduction of thickness [58]	
		ventriculomegaly [12,58]	
	cortical development	polymicrogyria, pachygyria, periventricular heterotopia [56,58,59]	
		focal cortical dysplasia [4,58]	
	cerebellar	hypoplastic or macro cerebellum [58,72]	
		absent or small cerebellar vermis [58]	
	other	CNS tumor [58,62]	
		large basal ganglia [58]	
		MC-I [15]	
epilepsy	seizures	febrile seizures [17,63,64]	AEDs (VPA; LEV; bi-therapy, tri-therapy [7,17,63]); VNS [65]; Steroids [63]
		focal seizures [17,63]	
		atypical absences [17,63]	
	EEG	focal abnormalities [17,63,64]	
		slowing of background activity [17,63,64]	
		ESES [63,64]	
sleep disorders	SDSC questionnaire	pathological results in “sleep wake transition” and “initiating and maintaining sleep” sub-scales [70]	melatonin; niaprazine, benzodiazepines [64,70]
	sleep architecture (polysomnography)	TST reduction and WASO increase [64]	

**Table 2:** Cognitive, developmental, and behavioral features in Mowat-Wilson Syndrome.

	Cognitive Characteristics	Behavioral and Developmental Characteristics		
Reference /Number of Patients	Cognitive Profile	Motor Development	Speech	Behavior
Mowat 1998 [73]/6	6/6 global developmental delay (severity not reported)	severe developmental delay	speech impairment, with some patients able to pronounce some words	-
Yamada 2001 [3]/10	10/10 severe intellectual disability	10/10 delayed motor development	speech impairment, some patients able to pronounce some words	-
Yoneda 2002 [78]/1	1/1 intellectual disability noted from 5 years	spastic paresis with hyperreflexia in the extremities	word comprehension almost normal, able to speak short sentences,	-
Mowat 2003 [4]/21	21/21 severe-moderate intellectual disability	global motor delay with median age of walking at 4, many non-deambulatory	speech is absent or restricted to a few words and is disproportionately delayed compared to comprehensionsome patients communicate successfully with signing	happy demeanor with frequent smiling
Zweier 2003 [8]/4	4/4 severe intellectual disability	-	-	happy affectionate in 3/4; not applicable in 1/4
Cerruti-Mainardi 2004 [9]/2	2/2 severe intellectual disabilityTest performed: *Denver II Scale*	2/2 motor developmental delay	2/2 speech impairment	-
Ishihara 2004 [10]/19	18/19 severe intellectual disability, 1 intellectual disability (mild phenotype, noted at 5 years)	18/19 delayed motor development	-	-
Adam 2006 [12]/32	all except a newborn have developmental delay	None of 3 patients < 24 months were ambulatory, 7 patient > 24 months, 1 walked at 2 years, 5/7 walked at 3 years, 1/7 walked at 8 years, wide based, (median age of ambulation at 3 years) or ataxic-like gait, fine motor skills were uniformly delayed	All patient > 1 years impaired verbal language skills, 5 pts have no spoken words	5 happy demeanors, 2 of these frequent laughter, 1 self-harm
Evans 2012 [75]/61	99% in severe-profound intellectual disability, not reported a single resulttest performed: *VABS, WISC-III IV, Denver scale, Griffiths*	-	-	unrealistically happy or elated, laugh or giggle for no obvious reason stand too close to others, high rate of oral behaviorsstereotyped behaviors; under reaction to pain, behavioral problems(assessed with *DBC)*
Kilic 2016 [77]/6	3/6 severe intellectual disability, 3/6 moderate intellectual disability	-	all presented speech impairment	All patients had happy demeanor and oral behaviors, like bruxism, mouthing, or chewing objects or body parts
Zweier 2006 [8]/1	1/1 not reported value, not severe-moderate	motor developmental delay	speech delay but by now he speaks in full sentences	-
Niemczyk 2017 [82]/26	-	-	-	incontinence, self-absorbed behavior(assessed with *DBC)*
Ivanovski 2018 [7]/87 (25 unpublished)	87/87 severe-moderate intellectual disability	hypotonia, developmental milestones delayed	speech impaired but with receptive language skills	-
Bonanni 2017 [63]/7	7/7 profound range of intellectual disability	-	absent speech	happy and sociable demeanor, repetitive and stereotyped behaviors
Ho 2020 [76]/15	15/15 severe-moderate intellectual disability	13/15 able to walk with ataxic gait	8/15 absent speech	8/15 cheerful and friendly demeanor
